# Effect of vortioxetine on proinflammatory cytokine levels in patients with heart failure and comorbid depression

**DOI:** 10.1192/j.eurpsy.2021.269

**Published:** 2021-08-13

**Authors:** A. Sikora, S. Fedorov, O. Pityk, M. Vynnyk

**Affiliations:** 1 Psychiatry, Narcology And Medical Psychology, Ivano-Frankivsk National Medical University, Ivano-Frankivsk, Ukraine; 2 Postgraduate Therapy, Ivano-Frankivsk National Medical University, Ivano-Frankivsk, Ukraine

**Keywords:** Depression, comorbidity, cytokines, heart failure

## Abstract

**Introduction:**

Several studies have shown impaired cytokine status in both patients with depression and chronic heart failure (HF).

**Objectives:**

to study the effect of vortioxetine on the level of pro-inflammatory cytokines: interleukin -1β (IL-1β) and interleukin - 6 (IL-6).

**Methods:**

there were examined 80 patients with HF with reduced ejection fraction (HFrEF) of ischemic genesis with functional class (FC) II-III (NYHA), 37 patients were without depression, 43 - with mild or moderate depressive disorders. Those with mild or moderate depressive disorders were divided into 2 subgroups: 21 patients received psychotherapy, 22 patients, in addition to psychotherapy, were prescribed vortioxetine at a dose of 10 mg / day in the morning after meals. The control group consisted of 20 healthy individuals. The level of cytokines in the blood was determined by ELISA method.

**Results:**

Patients with CHF have an increase in levels of pro-inflammatory cytokines. Thus, the concentration in the serum of IL-1β was 2.3 times higher than the same indicator in the control group: (56.45 ± 4.17) pg / ml, against (24.71 ± 4.21) pg / ml p <0.001). Depression caused an additional increase in the levels of IL-1β by 13.5% (p <0.05) and IL-6 - by 17.3% (p <0.01). Additional administration of vortioxetine caused a more rapid decrease in blood levels of both IL-1β (HR 0.87 [95% CI 0.72-0.97; p = 0.034]) and IL-6 (HR 0.81 [95% CI 0.68-0.93; p = 0.029]).

**Conclusions:**

Thus, vortioxetine causes a decrease in the concentration of pro-inflammatory cytokines IL-1β and IL-6 in patients with HF and comorbid depression.

**Disclosure:**

No significant relationships.

